# An assessment of AcquireX and Compound Discoverer software 3.3 for non-targeted metabolomics

**DOI:** 10.1038/s41598-024-55356-3

**Published:** 2024-02-28

**Authors:** Bret Cooper, Ronghui Yang

**Affiliations:** Soybean Genomics and Improvement Laboratory, USDA-ARS, Beltsville, MD 20705 USA

**Keywords:** Metabolomics, Mass spectrometry

## Abstract

We used the Exploris 240 mass spectrometer for non-targeted metabolomics on *Saccharomyces cerevisiae* strain BY4741 and tested AcquireX software for increasing the number of detectable compounds and Compound Discoverer 3.3 software for identifying compounds by MS^2^ spectral library matching. AcquireX increased the number of potentially identifiable compounds by 50% through six iterations of MS^2^ acquisition. On the basis of high-scoring MS^2^ matches made by Compound Discoverer, there were 483 compounds putatively identified from nearly 8000 candidate spectra. Comparisons to 20 amino acid standards, however, revealed instances whereby compound matches could be incorrect despite strong scores. Situations included the candidate with the top score not being the correct compound, matching the same compound at two different chromatographic peaks, assigning the highest score to a library compound much heavier than the mass for the parent ion, and grouping MS^2^ isomers to a single parent ion. Because the software does not calculate false positive and false discovery rates at these multiple levels where such errors can propagate, we conclude that manual examination of findings will be required post software analysis. These results will interest scientists who may use this platform for metabolomics research in diverse disciplines including medical science, environmental science, and agriculture.

## Introduction

Small molecules can be identified by a high-throughput method known as metabolomics^1^. A contemporary workflow includes extracting chemical compounds from biological or environmental samples, separating them by liquid (or gas) chromatography, measuring the mass-to-charge ratios (*m*/*z*) of the ionized compounds by mass spectrometry (the MS^1^ spectrum), and measuring the fragments of MS^1^ ions in an MS^2^ spectrum^2^. The relative amount of a compound can be estimated from its MS^1^ ion observed over time while compound identity can be deduced by comparing fragmentation ions in an MS^2^ spectrum to reference spectra of known compound standards curated in spectral libraries. This workflow allows one to identify hundreds to thousands of compounds and ascertain their relative changes in abundance between samples in a controlled experiment designed with statistical accuracy.

There are few standardized metrics for achievement, success, accuracy, or competency for metabolomics compared to the genomics and proteomics sciences. Statistics-based confidence metrics for DNA sequencing base calling are longstanding^3^, RNA sequences can verify gene sequences^4^, and proteins can be identified using genome sequences^5^. But when yeast, for example, produces about 16,000 defined compounds from 31,624 defined biochemical reactions using just 6000 genes^6^, or when there are 100,000 metabolites in human cells with 25,000 genes^7^, it becomes clear that if a genome is a closed set of information, the metabolome—the true number of metabolites in any given cell at any time under any condition—remains indeterminate^8^. This makes it difficult to measure the completeness of a metabolomics study.

The Thermo Scientific Orbitrap Exploris 240 is a commercially available mass spectrometer designed for metabolomics research. With its high resolving power (240,000 at 200 *m/z*), parts-per-billion mass accuracy, and picomole sensitivity, the instrument accurately distinguishes and measures thousands of masses (rather, *m*/*z*) per second. AcquireX software coupled with the instrument assists in reducing background ion detection, focusing on ions from experimental samples, and digging deep into a sample to find rare ions. Post-acquisition Compound Discoverer version 3.3 (CD3.3) software also coupled with the instrument takes raw files for each sample analyzed, aligns and normalizes peak areas, further distinguishes background ions, searches reference MS^2^ spectral libraries for compound identification, and performs statistical analyses to assess relative compound accumulation differences between samples. Overall, the Exploris 240 system is a (near) turn-key solution for the performance of metabolomics, but it may require months of practice and application before novice users understand how to get the most out the system. The purpose of the following study is to better illustrate the capacity of AcquireX and CD3.3 software by analyzing a yeast extract on the Exploris 240. The information herein will help users understand the platform and provide a baseline for which others may gauge success when performing metabolomics on yeast or other biological materials.

## Methods

### Yeast metabolite extraction

Auxotrophic *Saccharomyces cerevisiae* strain BY4741 [American Type Culture Collection (ATCC; Manassas, VA, USA); strain designation ATCC 4040002; https://www.atcc.org/products/201388] harboring empty plasmid pAG425GPD-ccdB with the *LEU2* gene [Addgene (Watertown, MA, USA); catalog # 14154; https://www.addgene.org/14154/] was used in this research to minimize potential co-culture contaminants. Yeast was maintained on solid or liquid media comprising yeast nitrogen base, ammonium sulfate, glucose, and SC-LEU powder containing amino acids except for leucine (Sunrise Science Products, Knoxville, TN). Yeast colonies from solid medium were grown in liquid culture (70 mL) overnight at 28 °C at 200 rpm on a shaking platform until the optical density measured at 590 nm was between 0.7 and 0.8. Aliquots (10 mL) were distributed to 6 tubes and centrifuged at 3000 × g at room temperature to pellet the yeast cells. The tubes were decanted, and the cells were washed with phosphate buffered saline (pH 7.2) and recentrifuged. The washed yeast pellets were resuspended in 1 mL 50% methanol and transferred to tubes containing 0.5 mm glass beads (Omni International, Kennesaw GA) to which 0.5 mL chloroform was added. The tubes were run through a Qiagen PowerLyzer 24 bead mill (Qiagen, Hilden, Germany) 10 times at 5000 rpm for 20 s (cooled on ice for 3 min between cycles). Two matrix blank samples were prepared the same way by adding methanol/chloroform/water to tubes with beads but without yeast. After milling, the samples were incubated at  − 20 °C overnight and centrifuged at 12,000 × g. The water/methanol (polar) and chloroform/methanol (non-polar) phases were recovered separately and dried in glass vials under vacuum.

### Mass spectrometry

The six polar phase residues and two corresponding matrix blanks were separately resuspended in 100 µL 50% methanol/0.1% formic acid and the six non-polar phase residues and other matrix blanks were resuspended in 100 µL 95% acetonitrile/5% 10 mM ammonium acetate/0.1% formic acid. The six polar phase samples were combined to make a pool, and the six non-polar phase samples were combined to make a separate pool, as were the matrix blanks. These pooled biological samples served multiple purposes for technical replicate injections, MS^2^ generation, and Quality Control (QC). Residual insoluble particulate matter was removed by centrifugation at 12,000 × g. Five µL injections of the pooled samples and blanks were used in the subsequent procedure. Injected polar phase pool and corresponding blanks were separated on a 150 × 2.1 mm Hypersil GOLD VANQUISH column with 1.9 µm particles (Thermo Fisher Scientific) at 40 °C coupled to a Vanquish HPLC pump (Thermo Fisher Scientific) controlling a 10-min linear gradient from 0 to 95% acetonitrile and 0.1% formic acid at a flow rate of 0.2 mL per minute (after which the column was re-equilibrated in 0.1% formic acid for 5 min at a flow rate of 0.2 mL per minute). Eluent was electrosprayed at 3.5 kV positive polarity into an Exploris 240 mass spectrometer (Thermo Fisher Scientific). The instrument was calibrated externally at less than 1 parts-per-million (ppm) RMS deviation in the low and high mass ranges and with an internal mass calibrant during MS^1^ scanning. Sheath gas was 35, auxiliary gas was 7, and sweep gas was 1 (arbitrary units). The ion transfer tube temperature was 325 °C and the vaporizer temperature was 275 °C. Default charge state was 1 and the expected peak width was 6 s. Advanced peak determination, mild trapping, internal mass calibration, and AcquireX method modifications were enabled. AcquireX Deep Scan was used to create a background ion exclusion list from the matrix blank and an ion inclusion list from a polar phase pool injection. Default Deep Scan settings were used except that [M+H]^+1^, [M-H_2_O+H]^+1^, [M-NH_3_+H]^+1^, [M+ACN+H]^+1^, and [M+MeOH+H]^+1^ were preferred ions (corresponding with major ions associated with the solvent system) and isotopes of fragmented precursors were excluded. MS^1^ survey scans were performed on the matrix blank injection used to create the ion exclusion list and on the QC injection to create the ion inclusion list (MS^2^ analysis was not performed at this step). The MS^1^ scans were acquired in the Orbitrap at 120,000 resolution (Full Width Half Maximum) over a range of *m*/*z* 70–800*.* The RF lens was 70%, the AGC target was standard, the maximum injection time was 100 ms, and microscan was 1. Then, six injections of the polar phase pool QC were performed by AcquireX to generate MS^2^ spectra (ID files). For the IDs, MS^1^ survey scans were recorded in the Orbitrap at 60,000 resolution (FWHM) over a range of *m*/*z* 70–800. Monoisotopic precursor selection was enabled, the minimum intensity was 5000, charge states were filtered to 1, dynamic exclusion was set at auto, and target mass and targeted mass exclusions had 3 ppm mass windows. Twenty precursor ions per cycle were selected within a 1.0 Da isolation window and were fragmented by high energy collision-induced dissociation (30%, 50%, 70% normalized stepped collision energy), and their MS^2^ fragment ions were resolved in the Orbitrap at 30,000 resolution (FWHM) with standard AGC target, maximum injection time of 54 ms, and 1 microscan. After each ID injection, the *m*/*z* for resolved ions were automatically appended to the exclusion list for the subsequent injection. When the ID injections were completed, the original matrix blank was injected followed by an injection of the polar phase pool and QC, performed three times to create a dataset of 3 technical replicate sample injections. For these, MS^1^ survey scans were recorded in the Orbitrap at 120,000 resolution (FWHM) over a range of *m*/*z* 70–800, and the RF lens was 70% (MS^2^ analysis was not performed for these technical replicate and matrix blank injections). This entire procedure was repeated using the same samples but with the mass spectrometer operating in negative ion mode at  − 2500 V. The other settings were the same except that the AcquireX preferred ions were [M-H]^−1^, [M-H_2_O-H]^−1^, [M+FA-H]^−1^, and [M+HAc-H]^−1^.

Injected non-polar phase pool and corresponding blanks were separated on a 100 × 2.1 mm Accucore-150-Amide-HILIC column with 2.6 µm particles (Thermo Fisher Scientific) at 60 °C coupled to the Vanquish HPLC pump controlling a 10-min linear gradient from 95% acetonitrile/5% 10 mM ammonium acetate/0.1% formic acid to 50% acetonitrile/50% 10 mM ammonium acetate/0.1% acetic acid at a flow rate of 0.5 mL per minute (after which the column was re-equilibrated in 95% acetonitrile/5% 10 mM ammonium acetate/0.1% formic acid for 5 min at a flow rate of 0.5 mL per minute). Eluent was electrosprayed at 3.5 kV positive polarity into the Exploris 240 mass spectrometer using an internal mass calibrant. Sheath gas was 50, auxiliary gas was 10, and sweep gas was 1 (arbitrary units). The ion transfer tube temperature was 325 °C and the vaporizer temperature was 350 °C. Default charge state was 1 and the expected peak width was 8 s. Advanced peak determination, mild trapping, internal mass calibration, and AcquireX method modifications were enabled. The preferred AcquireX Deep Scan ions were [M+H]^+1^, [M-H_2_O+H]^+1^, [M-NH_3_+H]^+1^, [M+ACN+H]^+1^, and [M+NH_4_+H]^+1^. All other procedures and settings were the same as for the polar phase injections. The non-polar pool was also analyzed in negative ion mode as before, and the AcquireX preferred ions were [M-H]^−1^, [M-H_2_O-H]^−1^, [M+FA-H]^−1^, and [M+HAc-H]^−1^. These mass spectrometry data files can be retrieved from massive.ucsd.edu (MSV000092514).

### Compound Discoverer data analysis

Positive ion mode and negative ion mode polar phase and non-polar phase studies were analyzed separately with CD 3.3 (Thermo Fisher Scientific). The Input File node was used to submit the three files for the replicate injection and pooled phase QCs, the three corresponding matrix blank files, and the 6 corresponding ID files (but not the 2 files used for generating the initial exclusion and inclusion lists for the IDs). The Select Spectra node was used with open settings and a default 1.5 S/N threshold. The ChromAlign node was used to align chromatographic peaks in all files to a QC file. The Detect Compounds node was used with 2 ppm mass tolerance, 10,000 minimum peak intensity, at least 5 scans per peak, peak detection S/N threshold 1.5, remove baseline true, gap ratio threshold 0.35, max peak width 0.25, and compound detection of [M+H]^+1^, [M+ACN+H]^+1^, [M+MeOH+H]^+1^, [M+H-H_2_O]^+1^, [M+H-NH_3_]^+1^ ions for polar sample positive mode, [M-H]^−1^, [M+FA-H]^−1^, [M-H-H_2_O]^−1^ ions for polar sample negative mode, [M+H]^+1^, [M+ACN+H]^+1^, [M+H-H_2_O]^+1^, [M-NH_3_+H]^+1^, [M+NH_4_+1]^+1^ ions for non-polar sample positive mode, and [M-H]^−1^, [M+FA-H]^−1^, [M-H-H_2_O]^−1^, [M-H+HAc]^−1^ ions for non-polar sample negative mode. The Group Compounds node was used with 2 ppm mass tolerance, 0.25 min retention time (RT) tolerance, peak alignment true, and a peak rating filter threshold of 0 for a minimum of 0 files (the default peak rating is 4, but we were interested in all peaks so we could understand the limitations of the software). The Fill Gaps node was used with 2 ppm mass tolerance, the SERRF QC Correction node^9^ was used with 65% QC coverage (found in at least 2 of 3 files) to normalize the peak area results, max QC area RSD 30%, max corrected QC area RSD 25%, and correct blank files true, and the Mark Background Components node was enabled with max sample/blank 3. The Search mzCloud node was used to compare MS^2^ spectra from ID files with the HighChem HighRes algorithm to all compound classes at precursor mass and FT fragment mass tolerances of 10 ppm (default) and no other filters. The Search mzVault node was used with precursor mass and FT fragment mass tolerances of 10 ppm (default) with the HighChem HighRes algorithm and no other filters to compare MS^2^ spectra from ID files to the NIST_2020_MSMS High Resolution library and to a custom library of 20 amino acid standards created by us with the same instrumentation and settings. The Predict Compositions node was set at 2 ppm mass tolerance with element counts C90 H190 N10 O18 P5 S5. The Apply mzLogic and Apply Spectral Distance nodes were set with 2 ppm mass tolerances. Filtering of results was performed to limit background ions, include normalized areas and to require MS^2^ of preferred ions (Supplementary Table S1).

## Results

### General overview of software functions

We evaluated how AcquireX and CD3.3 would help us identify compounds from yeast extracts. We applied a simple preparation method for polar and non-polar compounds, but by no means was it our intention to identify all yeast metabolites with one method. Greater amounts of starting material may improve the detection of some compounds, but material amount minimums and optimal amounts are not a topic of this investigation. Furthermore, mass spectrometer settings greatly affect ion acquisition. Mass spectrometer setting optimization has been explored but is not a topic of this investigation^10,11^.

AcquireX and CD3.3 work in conjunction for comparative metabolomics analysis. An experiment should contain sufficient replicates for statistics^7^. Matrix blanks (tubes without any biological sample) should be processed alongside samples such that the matrix blank contains all background ions introduced by sample handling and processing. A separate QC sample needs to be prepared by pooling a small, equal volume of resuspended metabolite extract from each sample (except the matrix blanks). When AcquireX is used for sample acquisition, the matrix blank sample is used to create the initial background MS^1^ ion exclusion list (i.e., list of *m*/*z* values excluded from MS^2^ acquisition). Then a QC sample is used to create the initial MS^1^ ion inclusion list (i.e., list of *m*/*z* values included for MS^2^ acquisition). Subsequently, MS^2^ (ID files) are generated from the QC sample using the prior ion exclusion and inclusion lists as a guide. In the subsequent ID iterations, the previously identified ions are automatically added to the exclusion list, and MS^2^ are acquired from unique, less abundant ions. Afterwards, the matrix blank and the QC are injected alongside each set of sample replicates for MS^1^-only acquisition.

Mass spectrometry data files are then submitted to CD3.3. CD3.3 identifies MS^1^ ions across the files, calculates peak areas, and uses the QC files to align over time the chromatographic features of all samples (including the IDs and matrix blanks) and make subtle normalizations based on QC features. CD3.3 does not normalize to any single internal standard but rather normalizes across compound features in the dataset. CD3.3 also extracts MS^2^ spectra from the ID files, associates these with corresponding MS^1^ ions of the sample files within tolerances of parent ion mass and RT, and compares these MS^2^ spectra to references in spectral libraries. CD3.3 uses the matrix blank files to identify other potential background ions in the dataset that escaped the AcquireX exclusion process. CD3.3 output is a list of compound features, each with a measured parent ion *m*/*z*, its RT, its peak area (per sample), and, if found, its associated MS^2^ spectrum and match scores (among other types of data).

### Compound feature detection

**Figure 1:**
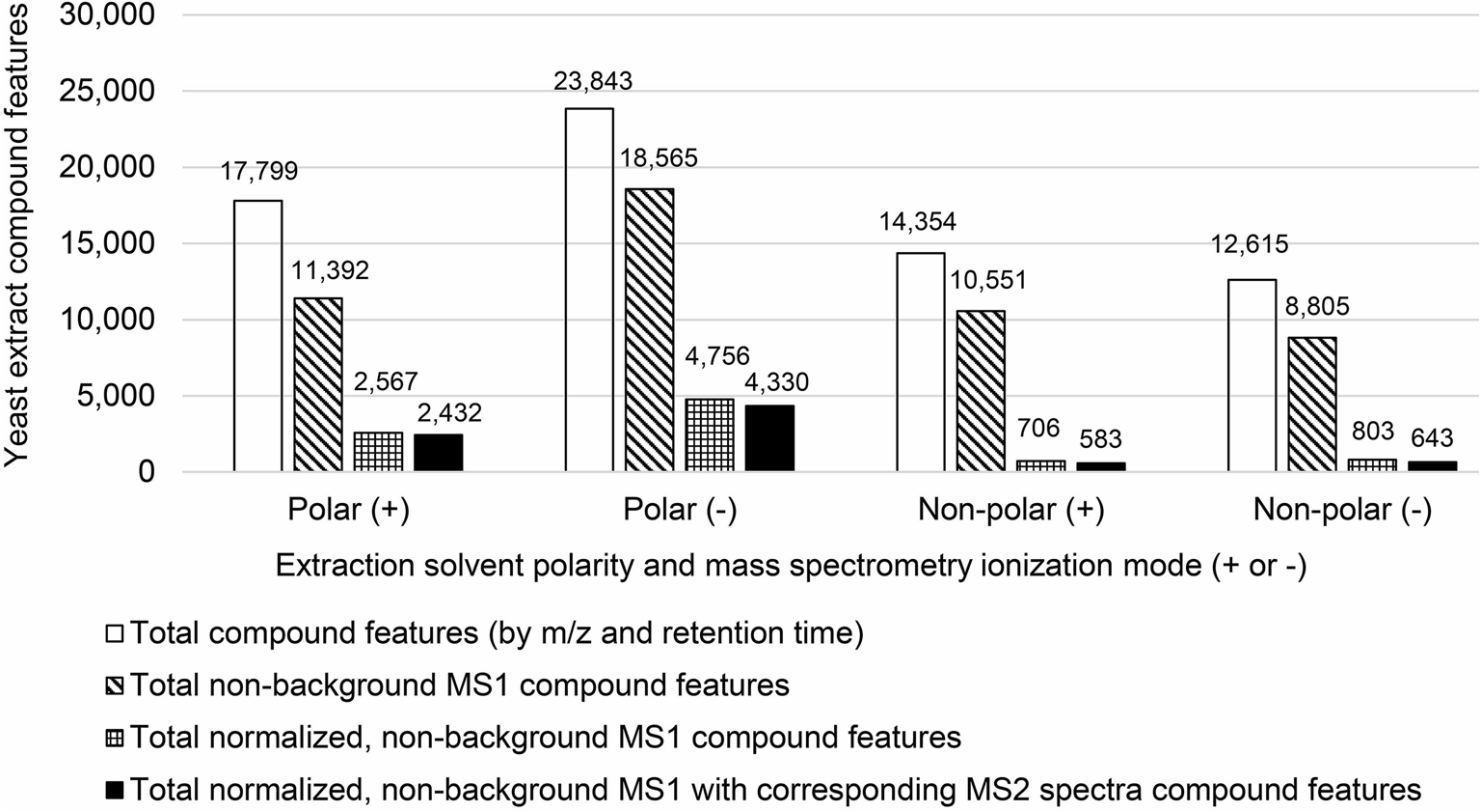
Compound features identified in polar and non-polar yeast extracts analyzed by positive and negative ion mode tandem mass spectrometry. Total number of compound features (MS^1^ ions by *m*/*z* and retention time) are compared to those filtered from background ions, those normalized and filtered from background ions, and those normalized with MS^2^ spectra and filtered from background ions. Polar extract solvents were methanol/water and non-polar extract solvents were chloroform/methanol.

There were 17,799 and 23,843 distinct compound features by *m*/*z* (MS^1^*)* and RT detected in the yeast polar fractions analyzed in positive and negative ion modes, respectively (Fig. 1). By contrast, fewer compound features were found in the non-polar fractions in both modes (Fig. 1). When CD3.3 filters were applied to the datasets to remove background ions and to normalize peak areas, most of these compound features, by at least several thousand, were eliminated from consideration. This demonstrates how the Mark Background Components and the SERRF QC Correction nodes function together to constrain weak data. Limiting the non-background and normalized MS^1^ parent ion features to those with corresponding MS^2^ spectra, there were 2432 and 4330 for polar fractions in positive and negative ion modes, respectively, and 583 and 643 for non-polar fractions in positive and negative ion modes, respectively (Fig. 1; Supplementary Table S1). These results revealed several general aspects of the system. (1) The Exploris 240 has high capacity to measure MS^1^ ions; its capacity is not a limiting factor for these samples. (2) Because the Exploris 240 capacity is so high, it will identify background ions even if AcquireX is used (this is not a reason to not use AcquireX; see following analysis). (3) The requirement for non-background, normalized ions with corresponding MS^2^ spectra produces a smaller but higher quality set of compounds with diagnostic fragment ion information.

The results also revealed that the negative ion mode generated nearly twice the number of non-background MS^1^ ions with corresponding MS^2^ spectra than the positive ion mode (Fig. 1). Comparing the calculated molecular weights and RTs of these compound features between the two ionization modes, we found a slight 7% overlap. This means that it is worth the effort to analyze samples in both positive and negative ion modes if the goal is to identify more compounds. Meanwhile, the non-polar fractions produced far fewer ions with corresponding MS^2^ spectra (Fig. 1). This implies that the non-polar fractions contained fewer or lower concentrations of metabolites. Nevertheless, unique ions were found in the non-polar fractions, with there being approximately a 1.8% overlap with the polar fraction based on compound feature calculated molecular weights. Hence, differential extractions with polar and non-polar solvents may be advantageous for certain applications.

### AcquireX

**Figure 2:**
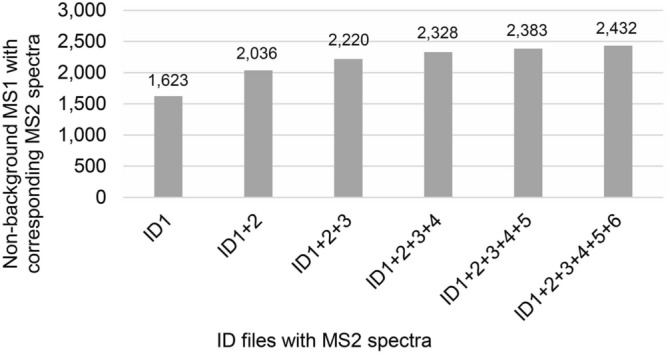
Total non-background MS^1^ ions with corresponding MS^2^ spectra found by adding consecutive AcquireX ID files containing MS^2^ spectra to the analysis.

When the Exploris 240 operated at 120,000 resolution (FWHM) for the MS^1^-only scans, it scanned from *m*/*z* 70–800 in about 250 ms and resolved hundreds to thousands of ions at any given RT. When producing MS^2^ spectra for the ID files, the Exploris 240 performed its MS^1^ scan twice as fast at 60,000 resolution (FWHM) but spent about a second and a half generating 20 MS^2^ spectra. In other words, the overall duty cycle increased due to MS^2^ spectrum acquisition. For each cycle, the 20 ions selected for fragmentation were only a small portion of the total available MS^1^ ions available for fragmentation and many relevant ions resolved in the MS^1^-only scans were not selected for MS^2^ fragmentation. AcquireX software attempts to rectify this and facilitate deeper MS^2^ acquisition by placing already analyzed ions on an exclusion list and performing multiple iterations of MS^2^ acquisition through repeated injections of the QC sample, the number of which can be defined by the user. We performed 6 injections of the QC pools with AcquireX, generating 6 ID files for each polar and non-polar fraction analyzed in positive and negative ion mode. We assessed the value of performing multiple ID iterations by recomputing the results from the polar fraction positive ion mode dataset, each time adding one more ID file to the analysis. About 84% (2036) of the normalized, non-background MS^1^ ions with corresponding MS^2^ spectra came from ID1 and ID2 files (Fig. 2). ID3 contributed 184 more with corresponding MS^2^ spectra, and ID4 contributed 105 more. ID5 and ID6 files contributed 55 and 49 more, respectively. Overall, the 5 additional ID acquisitions increased the number of normalized, non-background MS^1^ ions with corresponding MS^2^ spectra by 50%, but logical extrapolation implies that any further ID acquisition may have only provided a 2% gain. We examined the total number of MS^2^ spectra collected in files ID1 and ID6 and found 9411 and 7574, respectively. So, even though the ID6 file had fewer MS^2^ spectra, as might be expected, there were still thousands collected. Thus, the inherent function of AcquireX whereby it directs the mass spectrometer to fragment weaker and weaker parent ions over time likely explains the plateau in Fig. 2. We suspect that filters for CD3.3 or an inability to pair weak MS^2^ spectra with MS^1^ ions in sample files leads to fewer normalized, non-background MS^1^ ions with corresponding MS^2^ spectra being gained. So, while AcquireX indeed focuses instrument acquisition on more desirable ions over background, there is a limit to the gain. This can be empirically determined for any experiment as shown here.

### Compound identification by MS^2^ spectral library matching

**Figure 3:**
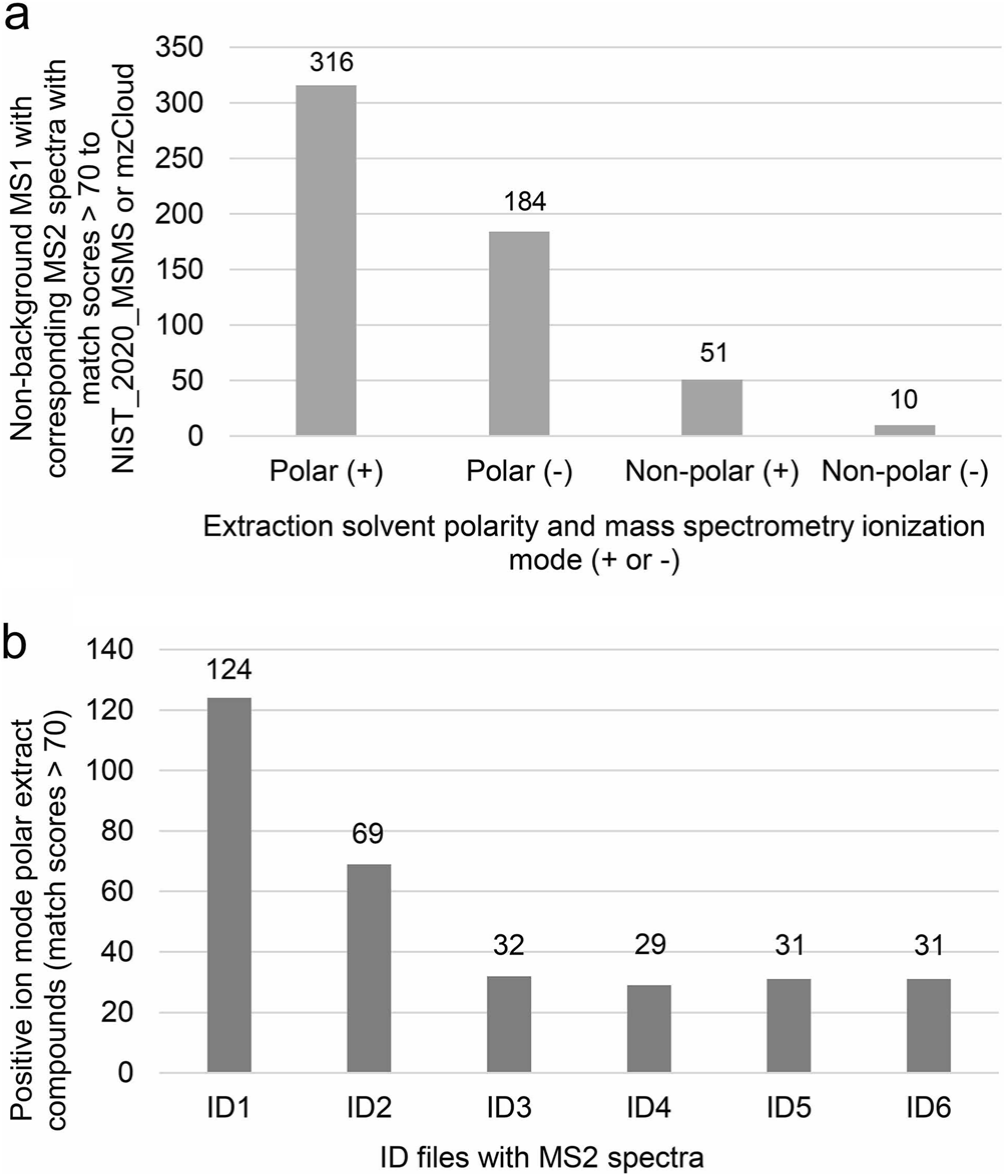
Non-background compounds identified by MS^2^ spectral library matching. (**a**) Number of non-background compounds from MS^1^ ions with corresponding MS^2^ spectra with match scores greater than 70 to a reference spectrum in NIST_2020_MSMS or mzCloud from polar and non-polar fractions analyzed in positive ( +) and negative (-) ion mode; (**b**) Number of non-background compounds from MS^1^ ions with corresponding MS^2^ spectra with match scores greater than 70 to a reference spectrum in NIST_2020_MSMS or mzCloud from polar fractions analyzed in positive ( +) ion mode per AcquireX ID file.

CD3.3 compared the observed MS^2^ to curated MS^2^ spectra from the NIST_2020_MSMS high resolution library, the mzCloud on-line library, and our custom library of 20 amino acid standards. One deficiency of the metabolomics field is the lack of straightforward methods for estimating the probability that an MS^2^ library match is a false positive and estimating the rate of false discovery among the set of matches^12^. This is partly due to there not being a complete set of metabolomic reference MS^2^ spectra for any organism to which observed MS^2^ spectra from an organism must match. Without this capacity, it is difficult to establish a match score threshold that reflects an acceptable false discovery rate. Efforts to address this problem are ongoing. One pertinent study estimates that using scores of 70 or greater (when the score range is 0 to 100) could reflect a 1–5% false discovery rate^13^. Thus, we accepted compounds with match scores greater than or equal to 70. Manual examination of all potential matches also was performed to resolve situations where the top score was not the best match due to observed parent mass and reference parent mass differences, to resolve ambiguity of multiple molecular formula predictions, and to resolve which match should characterize the ion when there were multiple, qualified matches to different compounds from different spectral libraries (examples follow). With these determinations, we found nearly twice as many high-scoring identifications from the polar fraction analyzed in positive ion mode than the negative ion mode (Fig. 3a) even though there were more MS^2^-identifiable compounds generated in negative ion mode (Fig. 1; Supplementary Table S1). This may be due to fewer negative ion mode MS^2^ spectra for compounds in the reference libraries. Furthermore, there were far fewer high-scoring identifications made from the non-polar fractions (Fig. 3a; Supplementary Table S1). Again, a potential lack of non-polar compound reference spectra could explain the reduced number of identifications, especially for ions collected in negative ion mode. As for the effect of AcquireX on compound identification, more MS^2^ spectra for the identified compounds came from the ID1 and ID2 files (Fig. 3b). After ID2, subsequent iterations added roughly 30 more compounds with high-scoring matches. Files ID3-6 combined, however, contributed to more than one-third of the total number of identifications. The union of all confident identifications from the polar and non-polar fractions analyzed in both positive and negative ion modes produced a non-redundant dataset of 483 compounds uniquely defined by their parent ion *m*/*z,* RT, and high scoring MS^2^ spectral library match.

### Examination of putative compound identifications

Among the set of putatively identified non-redundant compounds were 17 of 20 natural amino acids. Although the growth medium contained amino acids, we separated the yeast cells from the growth medium by centrifugation and washed the growth medium from the cells. On the additional basis of the robustness of their chromatographic peaks over background, we believe that the 17 amino acids detected were produced inside the yeast. The data that describe the detection of these 17 amino acids, and one of the amino acids not found, help explain how CD3.3 functions. We present six examples to illustrate common scenarios that CD3.3 users may encounter. Knowing what happens during data processing, why it happens, how it affects results, and how the results should be interpreted is critical to understanding metabolomics findings.

**Figure 4:**
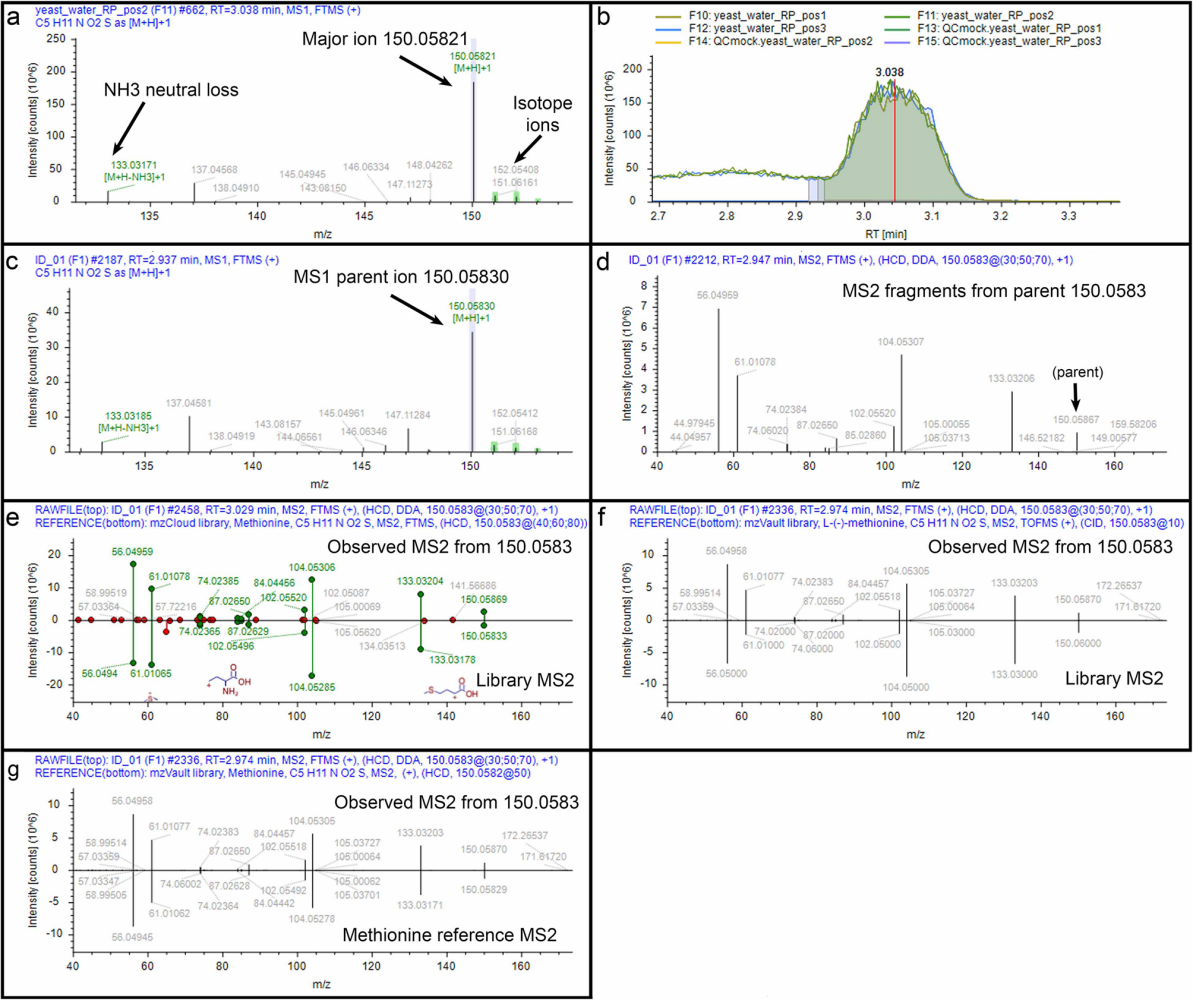
Example 1. (**a**) MS^1^ spectrum for detected ion *m*/*z* 150.05821 in a sample file; (**b**) Chromatographic representation of *m*/*z* 150.05821 ion from all sample files; (**c**) Corresponding MS^1^ spectrum for associated *m*/*z* 150.05830 ion in the ID file that is aligned to the analogous in the sample file in (a); (**d**) Corresponding MS^2^ spectrum from the ID file from preceding parent MS^1^ *m*/*z* 150.0583 ion from the ID file; (**e**) Mirror plot of match (score 96.0) between fragment ions of MS^2^ spectrum from parent MS^1^ *m*/*z* 150.0583 ion (top) and reference MS^2^ spectrum for methionine in mzCloud (bottom); (**f**) Mirror plot of match (score 97.3) between fragment ions of MS^2^ spectrum from parent MS^1^ *m*/*z* 150.0583 ion (top) and reference MS^2^ spectrum for methionine in NIST_2020_MSMS (bottom); (**g**) Mirror plot of match (score 94.1) between fragment ions of MS^2^ spectrum from parent MS^1^ *m*/*z* 150.0583 ion (top) and reference MS^2^ spectrum for methionine from a custom spectral library (bottom).

Example 1 (an ideal situation): CD3.3 found a major ion *m*/*z* 150.05821 at RT = 3.038 min. from the yeast_water_RP_pos2 file, the second injection of the polar fraction analyzed in positive ion mode at the MS^1^-only level (Fig. 4a). The software identified three corresponding ^13^C isotopic ions and marked them with green boxes to demonstrate they were within the expected mass range and amplitude. The software also found an NH_3_ neutral loss ion *m*/*z* 133.03171 that may have been produced during ionization (detection for this neutral loss was prescribed in the settings). Using the major *m*/*z* 150.05821 ion, the software evaluated the amplitudes of that ion over chromatographic separation time in each submitted file analyzed at the MS^1^-only level and calculated a peak shape and an area under the curve (Fig. 4b; baseline resolution did not occur for this peak and settings can be manipulated to dictate where chromatographic peak areas can begin and end). The software then found, within tolerances prescribed in the software settings, an *m*/*z* 150.05830 ion at RT = 2.937 min. in file ID1 (Fig. 4c) and an associated MS^2^ spectrum generated from that ion (Fig. 4d). Indeed, several associated MS^2^ spectra were generated during the elution time defined by the chromatographic peak. One of those MS^2^ spectra (Fig. 4e, top of the mirror plot) matched a methionine reference spectrum (Fig. 4e, bottom of the mirror plot) from mzCloud with a score of 96.0. Another MS^2^ spectrum matched a methionine reference spectrum from NIST_2020_MSMS with a score of 97.3 (Fig. 4f) and from our custom amino acid library with a score of 94.1 (Fig. 4g). Under our routine conditions, a methionine reference standard has RT = 3.07 min. Thus, these data substantiate the identification of methionine in yeast. This would be considered a Level 1 identification by the Metabolomics Standards Initiative^14,15^. Note, the subsequent examples are provided to demonstrate difficulty that arises with Level 2 identifications—those with MS^2^ match scores greater than 70 but without independent confirmation with a chemical reference standard.

**Figure 5:**
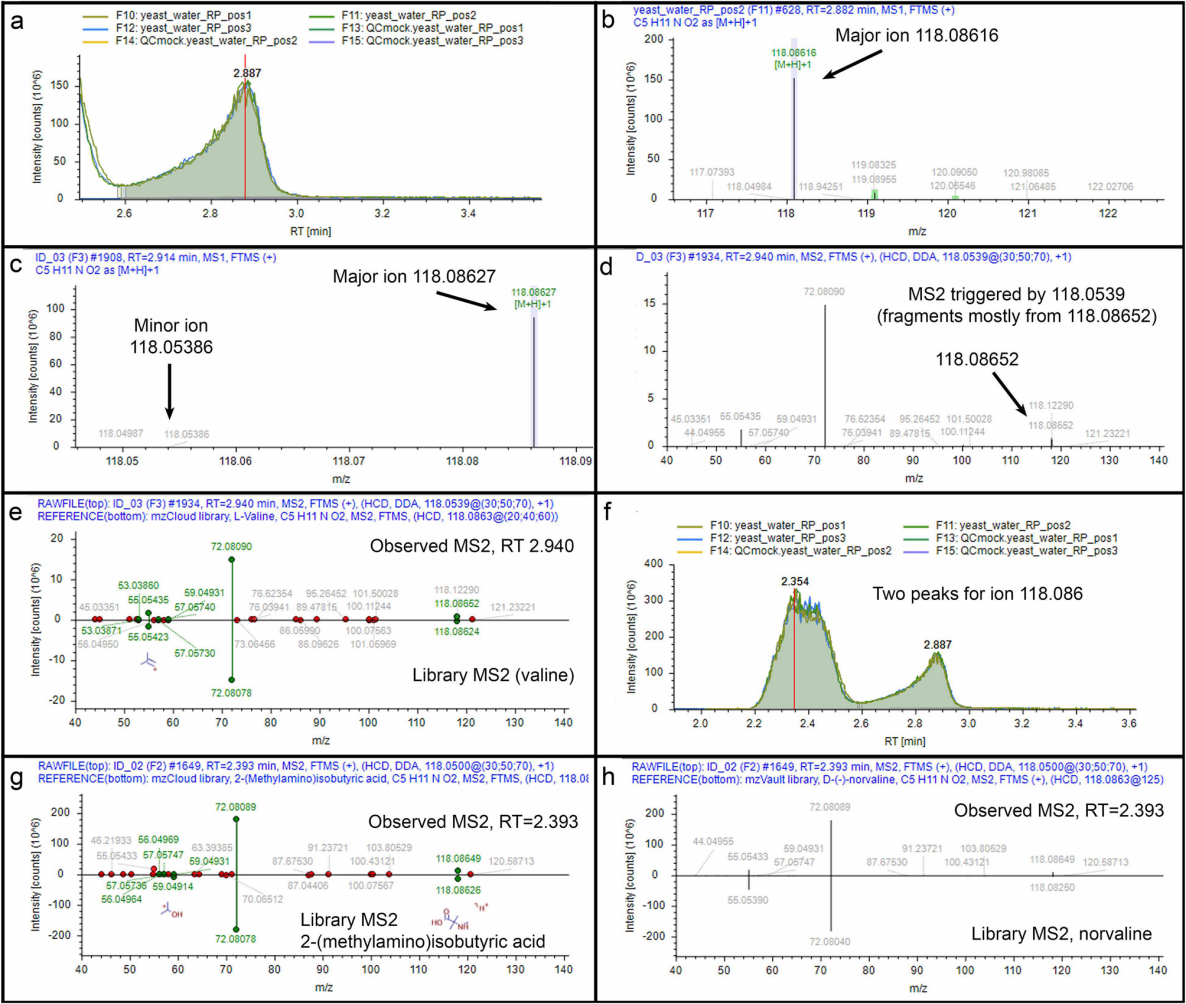
Example 2. (**a**) Chromatographic representation of *m*/*z* 118.08616 ion; (**b**) MS^1^ spectrum for detected ion *m*/*z* 118.08616; (**c**) Corresponding MS^1^ spectrum for associated major *m*/*z* 118.08627 ion and minor *m*/*z* 118.05386 ion; (**d**) Corresponding MS^2^ spectrum from preceding parent MS^1^ *m*/*z* 118.0539 ion; (**e**) Mirror plot of match (score 88.9) between fragment ions of MS^2^ spectrum from parent MS^1^ *m*/*z* 118.0539 ion (top) and reference MS^2^ spectrum for valine in mzCloud (bottom); (**f**) Chromatographic representation of two peaks for ion *m*/*z* 118.086; (**g**) Mirror plot of match (score 83.7) between fragment ions of MS^2^ spectrum from parent MS^1^ *m*/*z* 118.0500 ion (top) and reference MS^2^ spectrum for 2-(methylamino)isobutyric acid in mzCloud (bottom); (**h**) Mirror plot of match (score 90.8) between fragment ions of MS^2^ spectrum from parent MS^1^ *m*/*z* 118.0500 ion (top) and reference MS^2^ spectrum for norvaline in NIST_2020_MSMS (bottom).

Example 2 (co-fragmentation, mixed spectrum, isomers with the same match): CD3.3 found ion *m*/*z* 118.08616 with a chromatographic peak at RT = 2.887 min. (Fig. 5a and b) in the yeast_water_RP_pos2 file and found a matching ion in the ID3 file (Fig. 5c). The corresponding MS^2^ spectrum, however, was generated from a nearby *m*/*z* 118.05386 ion of weaker amplitude in its preceding MS^1^ spectrum (compare Fig. 5d to c). Although this MS^2^ spectrum appears to be mismatched to the first precursor, inspection of the MS^2^ reveals the presence of *m*/*z* 118.08652 (Fig. 5d). What happened? The Exploris 240 sufficiently resolved these two ions, but the isolation width was 1 Da, meaning that when the mass spectrometer tried to acquire the weaker *m*/*z* 118.05386 ion and fragment it, it co-isolated some of the dominant *m*/*z* 118.08652 ion and fragmented it as well. Thus, the MS^2^ spectrum contained a mixed population of ions whereby the dominant ion yielded the major fragments. Nevertheless, CD3.3 sorted this out in its processing and matched these dominant ions to a MS^2^ spectrum for valine in mzCloud with a score of 88.9 (Fig. 5e). Hence, the MS^2^ spectrum, despite being triggered by a different parent, was correctly assigned to the *m*/*z* 118.08616 ion, which matches the molecular mass of valine within  − 0.8 ppm. Interestingly, this ion also appeared at an earlier time point, RT = 2.354 min. (Fig. 5f). Under our conditions, purified valine has an RT = 2.89 min. Thus, the earlier peak likely represents a valine isomer. What is troublesome, however, is that the MS^2^ spectrum of the earlier peak also matched valine in mzCloud but with a higher score of 93.0! The next best matches were to 2-(methylamino)isobutyric acid in mzCloud with a score of 83.7 (Fig. 5g) and norvaline in NIST_2020_MSMS with a score of 90.8 (Fig. 5h). We provide this example to point out that 1) a researcher should verify MS^2^ assignments to precursors and look for precursor *m*/*z* evidence that supports a correct assignment, and 2) the top score might not always indicate the most correct match.

**Figure 6:**
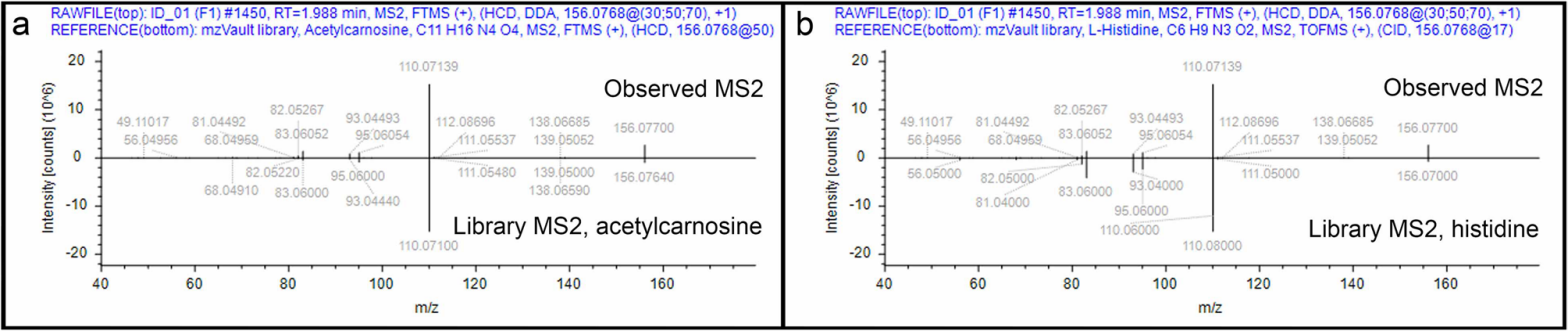
Example 3. (**a**) Mirror plot of match (score 96.6) between fragment ions of MS^2^ spectrum from parent MS^1^ *m*/*z* 156.0768 ion (top) and reference MS^2^ spectrum for more massive acetylcarnosine (268.1172 Da) in NIST_2020_MSMS (bottom); (**b**) Mirror plot of match (score 95.8) between fragment ions of MS^2^ spectrum from parent MS^1^ *m*/*z* 156.0768 ion (top) and reference MS^2^ spectrum for histidine (155.0694 Da) in NIST_2020_MSMS (bottom).

Example 3 (top score has an incorrect mass): An MS^2^ spectrum from the ID1 file matched acetylcarnosine in NIST_2020_MSMS with a score of 96.6, but the mass for acetylcarnosine is 113 Da higher than molecular weight of the parent ion of *m*/*z* 156.0768 (Fig. 6a). This was evident after manual examination of the matches whereby CD3.3 reported the Da deviation (ΔMass) for each compound matched. The second-best match with a score of 95.8 was to histidine with a reported mass deviation of  − 0.36 ppm (Fig. 6b). Histidine also was matched in mzCloud with a top score of 95.0. What happened here? The NIST search algorithm found sufficient spectral similarity of the fragment ions for acetylcarnosine, a more massive compound that has underlying structural similarity to histidine. Structural similarity information is useful when trying to identify compounds not represented in the libraries, but the appearance of good matches to compounds with greatly deviant masses means that manual examination of matches to the NIST_2020_MSMS library is required. Corroborating information such as matches to mzCloud can be used to resolve ambiguity when it appears.

**Figure 7:**
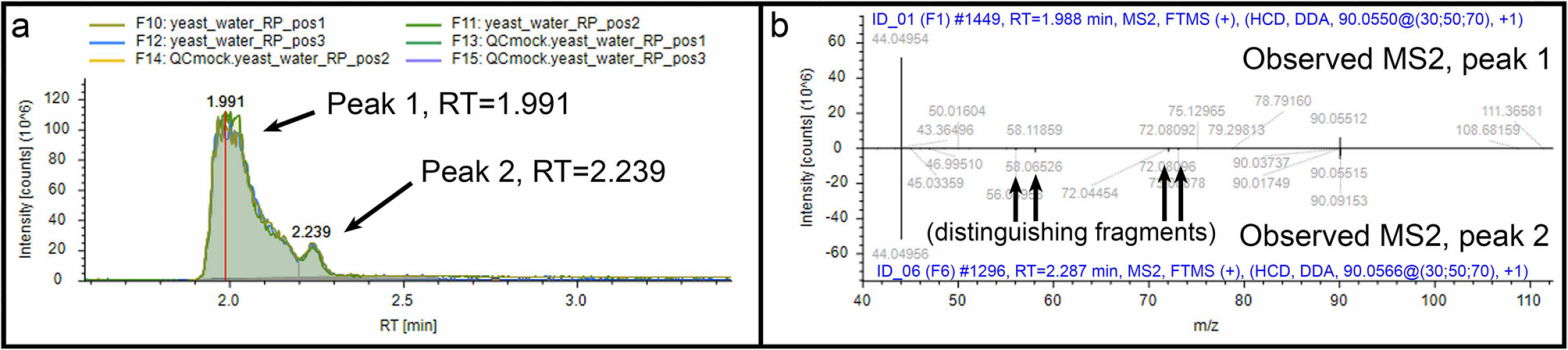
Example 4. (**a**) Chromatographic representation of two peaks for ion *m*/*z* 90.055; (**b**) Mirror plot comparison between MS^2^ spectrum from peak 1 (top) and MS^2^ spectrum for peak 2 (bottom).

Example 4 (separated adjacent peaks, top scoring matches are not the best matches): CD3.3 resolved two chromatographic peaks at RT = 1.991 min. and RT = 2.239 min. for *m*/*z* 90.05488 and 90.05493 ions, respectively, which match the molecular mass of 89.048 Da (Fig. 7a). Based on our alanine standard with an RT = 1.96 min., we know that the first peak at RT = 1.991 min. is alanine (Fig. 7a). Interestingly, the top hit from mzCloud to the spectrum assigned to that peak is alanine with a score of 78.9 while lactamide is the second-best match with a score of 78.5. By contrast, the top hit from NIST_2020_MSMS is sarcosine at 94.5 and the second-best match to alanine scores 76.1. To be sure, without routine experimental observation of alanine, we would otherwise assign this peak to the NIST_2020_MSMS top hit sarcosine, an isomer of alanine. The ion in the second peak, however, also resembles sarcosine and alanine. It likely is not alanine. CD3.3 has a feature to compare two MS^2^ spectra via a mirror plot. The MS^2^ spectrum from the second peak has minor ion fragments not observed in the alanine MS^2^ spectrum (Fig. 7b). Furthermore, the NIST_2020_MSMS match to sarcosine with a score of 84 is better than the match to alanine at 74. Thus, we would assign the second peak to sarcosine without having any better experimental evidence to determine otherwise.

**Figure 8:**
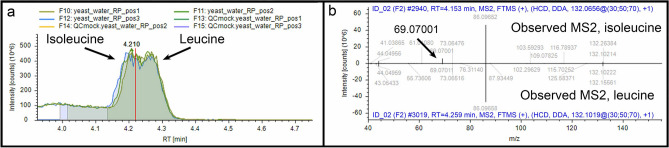
Example 5. (**a**) Chromatographic representation of peaks for isoleucine and leucine; (**b**) Mirror plot comparison between MS^2^ spectrum for isoleucine (top) and MS^2^ spectrum for leucine (bottom).

Example 5 (adjacent peaks not separated, different compound isomers merged as one): Of all amino acids we detected, we know that leucine was indeed made by yeast because the growth medium was devoid of leucine. Although leucine and isoleucine are isomeric, isoleucine separated before leucine at RT = 4.153 min. (Fig. 8a), and isoleucine fragmentation produced a characteristic *m*/*z* 69.07 ion (Fig. 8b). CD3.3, however, observed the two peaks as one, and it assigned the separate MS^2^ spectra for leucine and isoleucine to the merged chromatographic area. The points of this example are that 1) the calculated area of the curve can be a combination of two compounds (isoleucine and leucine in this case) if there is not sufficient separation between them, and 2) the spectra representing two isomeric compounds can get grouped with a single parent ion if their RTs fall within setting tolerances. This means that it may require manual examination of a peak and compound identification to tease apart isomers. Specifically, the RT tolerance setting in the Group Compounds node affects how adjacent peaks are distinguished and should be estimated for each experiment on the basis of observed retention drift.

**Figure 9:**
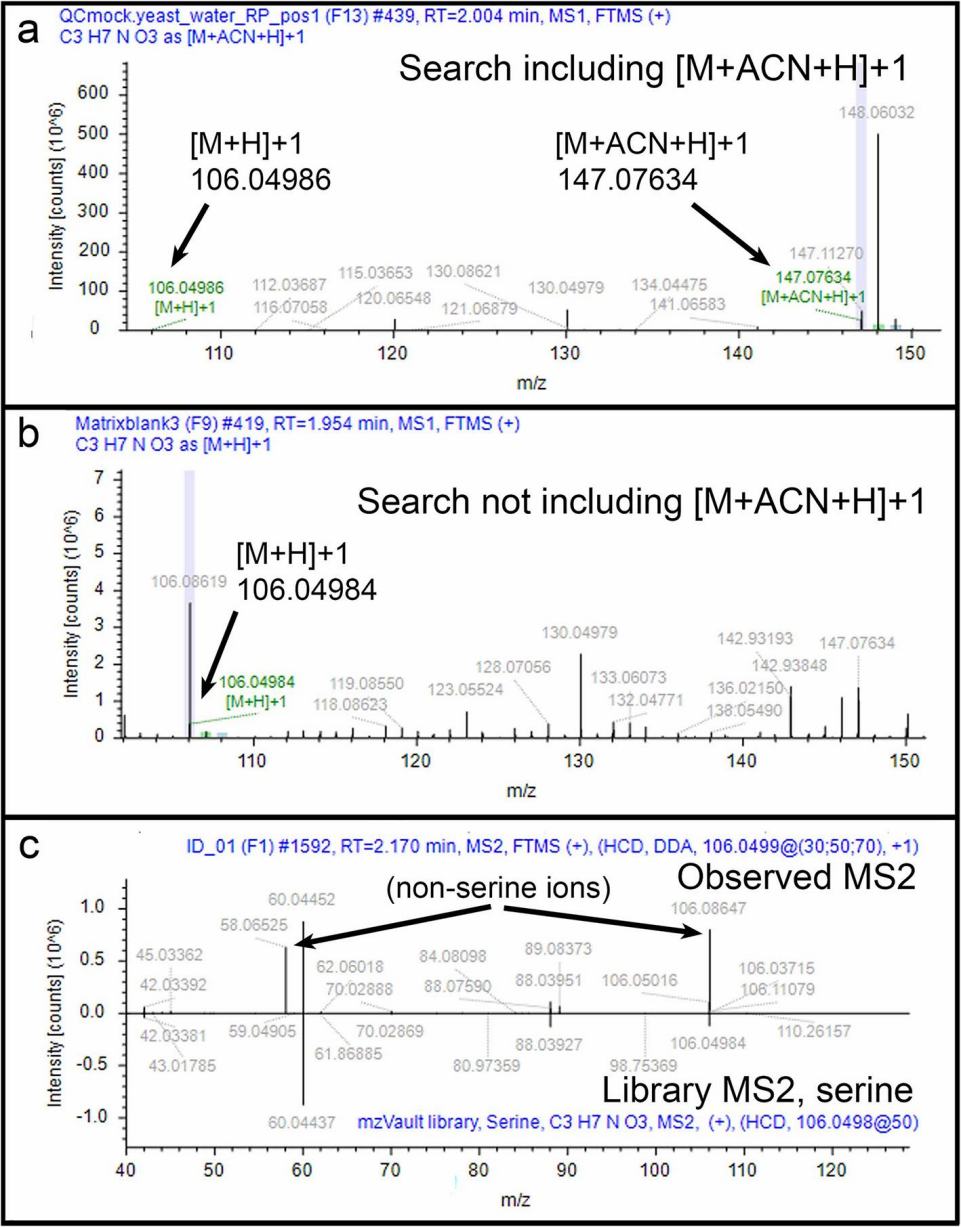
Example 6. (**a**) MS^1^ spectrum for detected [M+H]^+1^ *m/z* 106.04986 ion and associated [M+ACN+H]^+1^ ion when the CD3.3 Detect Ions node included a search for [M+H]^+1^ and [M+ACN+H]^+1^ ions; (**b**) MS^1^ spectrum for detected [M+H]^+1^ *m/z* 106.04984 ion (but not the [M+ACN+H]^+1^ ion) when the CD3.3 Detect Ions node included a search for only the [M+H]^+1^ ion; (**c**) Mirror plot of match (score 69.7) between fragment ions of MS^2^ spectrum from parent MS^1^ *m*/*z* 106.0499 ion (top) and reference MS^2^ spectrum for serine in NIST_2020_MSMS (bottom).

Example 6 (an expected compound is missing): Serine was one of the three amino acids that we did not identify in the yeast extracts. The serine molecular mass of 105.043 Da was not among the lower scoring or non-scoring compounds. We removed the data filters and discovered that the normalization filter hid the finding; with the filter removed, a molecular mass of 105.04253 Da appeared at RT = 2.017 min. We routinely find serine at RT = 1.90 min., so we looked closer at the associated MS^1^ spectrum and found that CD3.3 used the [M+ACN+H]^+1^ ion of *m*/*z* 147.0763 for generating the chromatographic peak instead of the [M+H]^+1^ ion of *m*/*z* 106.04986 because the former ion had a dominant amplitude (Fig. 9a). The resulting chromatographic peak areas from this ion, however, exhibited variation evident by a 75% RSD QC area. Software search settings for normalization restricted the RSD QC area to 30%, so this is why the finding was filtered. We tested what might happen if we did not include [M+ACN+H]^+1^ or any other ion except [M+H]^+1^ in the Detect Ions node. Under this condition, 105.04257 Da appeared in the results when using the normalization filter. This is because CD3.3 associated no other ion variants with the *m*/*z* 106.04984 ion (Fig. 9b). Consequently, the RSD QC area generated from this ion was 9% and passed the filter. The associated MS^2^ spectrum matched serine from our amino acid reference library but with a score of 69.7. The match score likely was suppressed by the *m*/*z* 58.06525 ion that may have been generated from a co-isolated *m*/*z* 106.08619 ion (Fig. 9c). The point of this example is to demonstrate that ion detection settings in the Detect Ions node can lead to false negatives and false positives. While this may be true to some extent for any setting, it will be difficult to know the rate at which this occurs for these settings when evaluating unknowns. Users will take little comfort with the prospect that if they only assign detected ions to [M+H]^+1^, then true ions like [M+ACN+H]^+1^ may be misassigned to the wrong compound (or not used when they should be). Similarly, if settings are open for the detection of all ions, then it is possible, for example, that CD3.3 may mistake a true [M+H]^+1^ ion of one compound as the neutral loss of another compound. Users are encouraged to evaluate these possibilities thoroughly.

## Discussion

Analyzing yeast polar and non-polar fractions in positive and negative ion mode, we identified a non-redundant set of 483 compounds with MS^2^ spectra (Fig. 3a). The identification of about 60% of these compounds can be attributed to AcquireX software that facilitated novel ion acquisition (Fig. 3b). Although scientists have performed metabolomics research on yeast for years, few have used MS^2^ spectra for compound identification^16,17^. Perruchon et al.^18^ identified about 50 compounds with MS^2^ spectra from *S. cerevisiae* using a mass spectrometer that does not collect MS^2^ spectra as quickly as the Exploris 240. Rampler et al.^19^ found 206 metabolites from *Pichia pastoris*, another yeast species, on a Q-Exactive (an older generation Orbitrap) using CD3.1 (an older version). These authors accepted some match scores lower than 70 or no score at all, and they did not search mzCloud or NIST_2020_MSMS. Although directly comparable yeast metabolomics studies are lacking for us, we hope that our study may be used for such purposes by others.

According to the Yeast Metabolome Database (YMDB), there are at least 16,042 small molecules in yeast^6^, 3915 of which are within our *m*/*z* range of detection. We estimate that we found about 11% of those subset YMDB compounds. This appears to be low coverage of the metabolome, but the reality is that not all yeast compounds have reference spectra in mzCloud and NIST_2020_MSMS, not all observed yeast MS^2^ spectra can be correctly assigned to a reference spectrum, and not all compounds were present in or extracted from our samples. This is a truism for all non-targeted metabolomics where the common lament is that too often only 10% of collected information is matched to compounds^20^. Hence, researchers are trying to get more out of the data their instruments acquire. Computerized algorithmic and machine learning approaches are being used to assign spectra to compound structures or predict compounds structures from spectra^21,22^. Other approaches include associating spectra based on evidence for plausible biochemical transformations of compounds. “Molecular networks” is a general term for workflows and algorithms that operate from such an approach of molecular connectivity^23–25^. In this way, Chen et al.^26^ identified 931 known metabolites and 686 putative metabolites from 5588 non-background ion peaks from negative ion mode yeast data. Although Chen et al.^26^ did not require MS^2^ spectra for all identifications like we have, we are within a comparable range of general mass spectrometry operation by distinguishing 4330 non-background ion peaks (but with MS^2^ spectra for negative ion mode data; Fig. 1). For us, it may be possible to use molecular connectivity to obtain more identifications after spectral library matching is exhausted. Indeed, CD3.3 has a Generate Molecular Networks node that establishes molecular connections based on biochemical transformations. We did not use that node in this study because we wanted to limit compound detection to spectral library matching. How the node increases yeast compound detection is worthy of future investigation. Some of the other ways to increase yeast compound detection can range from loading more sample, altering separation methodology, and manipulating the Exploris 240 settings. Meanwhile, the number of entries in mzCloud grows each month. It is likely that more yeast compounds can be matched from our data in the future.

Knowing how CD3.3 functions is crucial for successful compound identification. Here, we provide six examples of results a user may encounter with CD3.3 software. The first example is a Level 1 identification, a compound positively identified by a match to a purified standard analyzed on the same instrument (Fig. 4). In routine non-targeted metabolomics analysis, however, it probably is not possible to have reference standards for all detected compounds. So, most researchers will rely upon CD3.3 for Level 2 identifications of their compounds. The five other examples we provide regard such Level 2 identifications. Our examples reveal that sometimes the top scoring match is not to the true compound, that the same compound can get high scores at two different chromatographic peaks, that the top score can be made to a compound much heavier than the mass of the parent ion, and that MS^2^ spectra representing two different isomers can get grouped with a single parent ion (Figs. 5, 6, 7, 8 and 9). We can declare that the common thread to all of these examples, including the first example of an ideal identification, is that each required manual interrogation to sort out. Novices should know that accepting top score matches on a prima facie basis will lead to large numbers of incorrect matches and poorly-interrogated datasets of Level 2 identifications.

This brings us to a simple truth about the current field of metabolomics: it remains highly interpretative due to the limitations of compound identification by spectral matching. One such problem associated with this is that many software programs including CD3.3 that use spectral libraries for compound identification do not estimate the false positive rate for a MS^2^ match and do not estimate the false discovery rate for the set of matches. CD3.3 does perform statistical analyses and does calculate *p*-values and adjusted p-values (false discovery rates), but these calculations are for the chromatographic peak areas and have nothing to do with compound identification by spectral library matching. Users are advised to pay heed to this truth and not think that limiting CD3.3 data to the provided p-value and adjusted p-value cutoffs improves the certainty of compound identification. Notwithstanding, the lack of routine, accessible means by which false positive MS^2^ spectral matching and false discovery rates can be estimated (with respect to compound identification) hinders the entire field of metabolomics. Some contemporary approaches exist to address these needs^13,27^, but they are not integrated into CD3.3. Even if such metrics were provided, however, this would only solve one problem. Our examples reveal several other independent situations outside of spectral matching where false positives can arise. Many of these situations remain unmodeled.

We conclude by stating that one of the great benefits of CD3.3 is that it reveals multiple layers of data, thus allowing users to deeply investigate results. While such manual interrogation lends itself to subjectivity, it allows users to evaluate evidence for discovery at multiple levels. This is appropriate because we believe that when it comes to compound identification for a comparative study, multiple levels of proof are usually required^15^.

### Supplementary Information

Supplementary Table S1.

## Data Availability

Mass spectrometry data files can be retrieved from massive.ucsd.edu (MSV000092514).

## Abbreviations

*m*/*z*: Mass-to-charge ratio
CD3.3: Compound Discoverer version 3.3
ppm: Parts-per-million
ID: Identification
RT: Retention time
QC: Quality control
YMDB: Yeast Metabolome Database
